# Temperature-Dependent and Semi-Quantitative Enzyme Profiles of *Malacosoma disstria* (Lepidoptera: Lasiocampidae) Hemocytic Cell Lines

**DOI:** 10.3390/cells15030302

**Published:** 2026-02-05

**Authors:** Paschalis Giannoulis, Helen Kalorizou

**Affiliations:** 1 Department of Natural Resource Sciences, McGill University, Macdonald Campus, 21111 Lakeshore Road, Sainte-Anne-de-Bellevue, QC H9X 3V9, Canada; 2 Department of Agriculture, Faculty of Agricultural Sciences, University of Patras, New Buildings, 30200 Missolonghi, Greece

**Keywords:** lepidopteran hemocytes, insect tissue culture, physiological profile, cellular responses, bacterial challenge

## Abstract

**Highlights:**

**What are the main findings?**

- Ex vivo hemocyte populations derived from *Malacosoma disstria* (Lepidoptera: Lasiocampidae) demonstrated the capacity to preserve select temperature-dependent characteristics observed under in vivo conditions; however, notable variations in cellular morphology and population frequency per hemocyte type were evident when compared with their natural physiological state.
- Established hemocytic cell lines from *M. disstria* exhibit the ability of autonomous innate immune responses independent of systemic regulatory influences present in vivo, with such responses being readily detectable using semi-quantitative methodologies.

**What are the implications of the main findings?**

- The floating and adherent polystyrene-cultured cell lines derived from *M. disstria* demonstrate distinctive physiological and immunological properties, establishing a valuable experimental framework for elucidating innate immune mechanisms in invertebrates.
- Semi-quantitative approaches for hemocytic cell line enzymatic detection can yield valuable insights concerning the temporal dynamics governing enzyme release, thereby establishing a foundation for elucidating the kinetic parameters that drive cellular processes.

**Abstract:**

Insect hemocytic cell lines offer substantial advantages over primary, in vivo hemocyte cultures, fundamentally transforming experimental approaches in cellular immunology and related fields. Selected *Malacosoma disstria* cell lines were characterized for optimal growth temperatures, morphogenesis, blebbing, extracellular enzyme profiles, and their interactions with material (polystyrene) and microbial (*Bacillus subtilis*) surfaces. The adhesive hemocyte lines UA-Md221 and Md108 showed optimal growth at 28 °C, whereas UA-Md203 and Md66 grew best at 21 °C, with Md66 tolerating 21–28 °C. Md108 demonstrated a broader temperature tolerance than other adherent cultures. Both Md108 and UA-Md221 adhered to polystyrene within 24 h post-subculturing, although protease-induced morphological changes in modified Grace’s medium continued through 48 h and 72 h, respectively. Culture quality was monitored by assessing the release of multiple enzymes, including alkaline and acid phosphatases, esterases and lipases, aminopeptidases, proteases, glycosidases, and hydrolases from the cell lines at 50% confluency in modified Grace’s medium. Fetal bovine serum showed elevated esterase lipase (C8) and phosphoamidase activities when diluted in Grace’s medium and phosphate buffered saline (PBS). Exposure to dead *B. subtilis* suspended in PBS induced quantitative and qualitative alterations in the enzyme secretion profiles of Md66 and Md108 cultures. We conclude that semi-quantitative assessments of hemocytic cell lines can provide valuable insights for the time window of each enzyme release, revealing immune and metabolic signaling patterns.

## 1. Introduction

Innate responses to non-self-particulate antigens comprise phagocytosis, nodulation, and encapsulation [1]. These responses are initiated when foreign materials including microbial antigens [2], carboxy-modified latex, agarose, and dextran beads [3,4] adhere to immunocompetent hemocytes. Phagocytosis, the cellular mechanism for internalizing particulates [5,6], proceeds through antigen recognition and attachment, triggering signal transduction cascades that enhance pseudopod formation and culminate in antigen ingestion [7]. This ingestion phase is mediated by cytoskeletal regulatory proteins including D-SCAR, D-WASp, and profilin [8].

Nodulation represents the primary hemocyte response to bacterial populations and non-hyphal fungal structures in insects [9]. This process involves antigen-stimulated hemocyte aggregation, wherein foreign antigens become entrapped within granular cell-derived matrix proteins, followed by plasmatocyte encapsulation, ultimately facilitating the hemolymph clearance of microorganisms [1,10,11,12,13,14]. Surface binding of foreign materials triggers both phagocytosis and nodulation through cell signaling [15], culminating in extracellular matrix protein release from granular cells and spherulocytes [16,17,18,19].

Extracellular matrix proteins of insect blood cells including lacunin protein [20], noduler protein [9], and type IV collagen [21] enhance hemocyte adhesion to invaded microorganisms. Calreticulin, a 60 kDa endoplasmic reticulum protein, mediates adhesive and phagocytic activities, as demonstrated in *Pieris rapae* larvae responding to yeast cell challenge [22]. Polydnavirus infection of larval hemocytes in the lepidopteran fall armyworm, *Spodoptera frugiperda*, suppresses calreticulin gene expression and subsequently reduces prophenoloxidase activity, immulectin-2 levels, and hemocyte scavenger receptor expression, thereby limiting the host’s immune responses [23]. Encapsulation in lepidopteran hemocytes is a similar mechanism to nodulation but accommodates larger foreign objects exceeding hemocyte dimensions [24]. Nodulation and encapsulation interfere with the acquisition of nutrients and oxygen by the trapped microorganisms, and as a result of phenoloxidase-mediated melanin deposition, the activities of other enzymes released from hemocytes, and enzyme-generated radical production, there is no proliferation of the microbes [24].

Adhesion of plasmatocytes to foreign surfaces can lead to apoptosis of granular cells evidenced by chromatin condensation, intranucleosomal DNA fragmentation, and cell blebbing [25]. Blebbing is a common response of insect hemocytes to bacterial toxins produced by entomopathogenic bacteria such as *Photorhabdus luminescens* subsp. *akhurstii* strain W14 [26] and polydnaviruses released during oviposition by the hymenopteran parasitoid wasp *Cotesia congregata* [27], and it is regarded as a major general stress response [28].

Foreign recognition is defined by lepidopteran humoral pattern recognition proteins binding to antigenic receptors [10] and by hemocyte receptors including RGD-integrin receptors on plasmatocytes [17,29]. RGD-integrin receptors bind to collagen type IV or their fragments generated by metalloproteinases released by invading bacteria [30] and to discharged granular cell extracellular matrix proteins [21]. Indirect recognition receptors are represented by Toll and Toll like (TLR) transmembrane receptors [31]. Serine proteases activated by pattern recognition receptors binding to antigens elicit proteolytic activation of the cytokine-like transducer Spätzle in Diptera and Lepidoptera species, which activates Toll [32]. Independently of Toll protein family function, the Imd pathway also senses the presence of Gram-negative bacteria in insect hemocytes [33]. The Imd pathway has been shown to share homology between lepidopteran and dipteran fat body cells [34]; however, whether similar homology exists at the hemocyte level across these two insect orders has yet to be conclusively determined [35]. Peptidoglycan recognition proteins (PGRPs), expressed both on hemocyte surfaces and as soluble factors in the hemolymph, differentially activate the Toll and Imd signaling pathways in a subtype-dependent manner [36]. Signaling in hemocytes in response to adhesion and engulfment of foreign particles may involve numerous molecules, for example, activation of mitogen-activated protein (MAP) kinases such as c-jun N-terminal (JNK) kinase and p38 mitogen-activated protein [15], recognition by the soluble form of Down Syndrome Cell Adhesion Molecule [37], and intracellular cascades linked to Toll transmembrane receptors [38]. Additional hemocytic signaling includes the availability and concentrations of ions such as Ca^+2^ [39], Fe^+2^ [40] and Zn^+2^ [41], GTPase function [42], PKA, and PKC [28,39] and re-modelling of actin [43,44].

Non-self-responses by hemocyte cell lines have been described for lepidopterans in the presence of microbial antigens. Antimicrobial nitric oxide derivates (e.g., nitrite and nitrate) are detected in the supernatant of the hemocyte cell line of the salt marsh caterpillar, *Estigmene acreae* BTI-EA-1174-A. when the cells are incubated with *E. coli* LPS or silica beads [45,46]. Exposure of the BTI-EA-1174-A cell line to *E. coli* LPS triggers substantial release of active proteases [47], suggesting that lepidopteran hemocytes may retain the capacity to detect microbes through surface antigens even when situated outside the hemocoel.

*Malacosoma disstria* (Lepidoptera: Lasiocampidae) is a major pest of North America’s tree species [48,49]. Like other lepidopteran hemocyte cell lines [50,51], *M. disstria* hemocyte lines have been developed to facilitate studies on gene expression, ecdysteroid action [52,53], polyploidy [54], and viral activity [55,56]. Unlike the innate cellular immunity, which is documented with fresh hemolymph [39,57], the *M. disstria* cell lines allow examination of such immunity without plasma and hence facilitate direct studies of hemocyte-antigen interaction. Most *M. disstria* cell lines vary in their adhesiveness to foreign surfaces [56] and thus possibly in their immune responses. The floating *M. disstria* hemocyte cell line, Md66, adheres to polystyrene flasks after 6 days post-treatment with the molting hormone 20-hydroxyecdysone, by producing cytoplasmic projections [52]. Fragments of Md66 cell line hormone receptor 2 (MdHR2) and 3 (MdHR3) cDNA show homology with the ecdysone-inducible *E75* gene of *M. sexta*, *G. mellonella*, and *D. melanogaster* and hormone receptor 3 of *M. sexta*, *G. mellonella*, and *D. melanogaster*, respectively [52]. In mosquitoes, the *E75* gene product affects indirectly insect immunity enhancing vitellogenin upregulation and subsequently the synthesis of antimicrobial defensins by fat body and release to hemolymph [58].

Adherent *M. disstria* hemocyte cultures demonstrate an extensive variation with respect to time for dissociation from polysterene surfaces [56,59]. Enzymatic treatment of Md108, UA-Md203, and UA-Md221 cell lines with proteases (trypsin with or without collagenase) causes release of adhering *M. disstria* hemocytes from the surfaces of tissue culture flasks [56,59]. Initial adhesion of insect hemocytes to foreign surfaces is physico-chemically influenced by hydrophobic [60] and electrostatic interactions between those surfaces and the hemocyte membranes [61]. In most studies of attachment by insect hemocytes, emphasis is placed on hemocyte receptors and extracellular matrix proteins. Protein families of integrins [29,62] and selectins [63], collagen formation [64], and immunoglobulin-containing molecules [65] have been implicated in adhesion process. Physicochemical analyses of integrins [66] and fibronectins indicate that they produce a positive electrostatic charge on the surfaces of various human cell types [67], mollusk hemocytes [68,69], and insect muscle cells [70]; this is in contrast to the negatively charged surface of polystyrene [71], and thus, cellular charge may influence the capacities of the hemocyte to attach to the latter. Adhesion to negative surfaces varies among insect species. For example, desert locust, *Schistocerca gregaria*, larval hemocytes, in absence of plasma, do not engulf negatively charged carboxyl methyl sepharose beads [72,73]; in contrast, hemocytes of the American cockroach, *Periplaneta americana*, adhere to and accumulate on the surfaces of such beads [72].

Polystyrene is an aromatic hydrocarbon styrene polymer [74,75]. Polystyrene in standard tissue culture flasks is treated with ionized gas in an electric field; this process makes the material surface more wettable, facilitating contact of biological material to the substrate [76]. Hydroxyl groups on polystyrene favor cell adhesion of human leucocytes through interactions with complement factor C3b [77,78]; however, the mode of action has yet to be delineated [78] since little is known of the ways in which the hemocytes interact with polystyrene.

*Bacillus subtilis* is a rod-shaped, aerobic, Gram-positive, endospore-forming bacterium commonly found in soil [79] and has been widely employed in immunological studies [12,80,81,82,83,84]. As a negatively charged bacterium, it serves as a useful biological particle for comparing hemocyte responses with those elicited by synthetic materials such as polystyrene.

*M. disstria* hemocyte innate cellular responses in vivo [57] and in vitro (as primary cultures) with bacteria and their surface antigens [39,85] have been studied. However, few studies have been conducted on the non-self-responses of the hemocyte cultures of *M. disstria* [86]. The integration of cellular analysis approaches spans multiple levels of complexity, each providing unique insights into cellular function [87]. The integration of cell morphology with enzyme release patterns reveals critical structure–function relationships and secretion mechanisms, while combining morphological analysis with physiological parameters elucidates adaptation mechanisms and morphological plasticity [88]. When morphology is studied alongside innate immunity, important insights regarding pathogen recognition and phagocytic processes emerge [89]. Similarly, examining enzyme release in conjunction with physiological states illuminates metabolic regulation and energy allocation strategies, whereas the coupling of physiology with immunity uncovers stress-immunity crosstalk and associated trade-offs [90]. More complex integrative approaches yield a deeper understanding of cellular systems. The most comprehensive approach involves the simultaneous examination of morphology, physiology, enzyme release, and immunity, enabling complete cell phenotyping through thorough characterization across all possible routes [91]. This holistic methodology supports the development of predictive models and establishes standardized protocols for quality control and cell line validation, providing a powerful framework for understanding cellular complexities and environmental responses.

This study determined optimal growth temperatures for adherent and floating *M. disstria* cell lines; characterized their extracellular enzyme profiles to ensure culture integrity; and investigated morphogenesis, cell type frequencies, and blebbing as a stress marker. Cell lines that closely resembled freshly isolated larval hemocytes were selected to examine non-self-responses to polystyrene and *B. subtilis*. These responses were assessed through enzyme profile analysis in phosphate-buffered saline at the optimal growth temperature.

## 2. Materials and Methods

### 2.1. Hemocyte Cell Lines

Four hemocyte cell lines of larval *M. disstria* hemocytes (UA-Md203, UA-Md221, Md108, and Md66) were used initially. UA-Md203 was derived from hemocytes of *M. disstria* larvae hatched from eggs collected from *Malus* sp. in Edmonton (Alberta) and UA-Md221 from eggs on *Populus tremuloides* in the Peace River district of Alberta [56]. Md108 (provided by Forestry Canada, Sault Ste Marie, ON, Canada) was derived from *M. disstria* larvae hatched from eggs found in the Sault Ste Marie area, and Md66 was derived from Md108 [54,59,92]. The culture medium was formulated based on Grace’s insect tissue culture medium (Gibco, Thermo Fisher Scientific, Burlington, ON, Canada) and was supplemented with bacto tryptose broth at a concentration of 0.25 g per 100 mL of medium (Difco, Becton, Dickinson and Company, Mississauga, ON, Canada) [59]. The pH was adjusted to 6.2 using potassium hydroxide (KOH, 1N) followed by sterilization through a 0.22 μm pore size filter. This medium was subsequently supplemented with sterile heat-inactivated fetal bovine serum at 56 °C for 30 min (Gibco, Thermo Fisher Scientific, Burlington, ON, Canada), with final concentration 8% *v*/*v*, and is referred to as culture medium throughout this study.

The cell lines Md108, UA-Md203, and UA-Md221, which grow adherent to the polystyrene substrate, were subcultured by dissociating the hemocytes upon reaching 80–90% confluency. The dissociation procedure involved removal of the culture medium followed by rinsing of the adherent cells with 5 mL of Rinaldini’s balanced salt solution (0.8 g NaCl, 0.02 g KCl, 0.1 g Na citrate, 0.005 g NaH_2_PO_4_, 0.1 g NaHCO_3_, and 0.1 g glucose in 100 mL deionized water) [93]. The cell lines UA-Md203 and Md108 were then incubated in 0.05% (*w*/*v*) trypsin in Rinaldini’s balanced salt solution for 1 h [56] and 5 min [59], respectively. The cells were subsequently rinsed with and finally suspended in culture medium (5 mL). For cell line UA-Md221, dissociation involved incubation for 1 h at 21 °C with both trypsin (as above) and collagenase (0.05%) [56]. This cell line was rinsed free of proteases using culture medium and suspended by gentle pipetting. Stock cultures were maintained by inoculating 25 cm^2^ Corning^®^ flasks (Thermo Fisher Scientific, Burlington, ON, Canada) with 2 × 10^4^ cells per 5 mL of culture medium and incubating at 21 °C in the dark. For the floating cell line Md66, 1 mL of cell culture at 80% confluency was added to 5 mL of fresh culture medium. For experimental purposes, cells from all four culture types were utilized upon reaching 50% confluency in tissue culture flasks, unless otherwise specified. This confluency level was selected because the hemocyte density per mm^2^ in tissue culture flasks approximated the total hemocyte counts observed in hemocyte monolayer assays using whole hemolymph from *M. disstria* [39], where adherent cells were analyzed.

### 2.2. Bacteria

Stock cultures of *B. subtilis* (Boreal Biologicals, St Catharines, ON, Canada) were subcultured every 2 weeks on tryptic soy agar and incubated at 5 °C. For experimental tests the bacteria were incubated at 25 °C in tryptic soy broth (2.5 g/L medium, Difco, 10 mL) in scintillation vials (20 mL) on a gyrotary shaker at 250 rpm (model G-10, New Brunswick Scientific, Edison, NJ, USA) until they achieved an optical density at 660 nm of 0.75. Dead bacteria were used to avoid ongoing bacterial metabolism from influencing results [94]. Bacterial inactivation was achieved through ultraviolet irradiation at 203 nm for 3 h (20 mL sample volume, 25 °C) using a Spectroline PL-265T lamp (Spectronics, Westbury, NY, USA). Cells were subjected to centrifuge washing, which consisted of three cycles of centrifugation (20 mL, 12,000× *g*, 2 min, 25 °C) followed by resuspension in PBS (1 mL). Viability was subsequently assessed by spreading 100 μL aliquots of the bacterial suspension onto tryptic soy agar plates. Following incubation for 96 h at both 25 °C and 30 °C, colonies were counted; the absence of colony formation was interpreted as confirmation of bacterial death.

### 2.3. Optimum Temperature for Hemocyte Growth

Flasks containing 10 mL of culture medium were inoculated with 4 × 10^4^ cells prior to incubation at selected temperatures. Because the ambient temperature experienced in the field by *M. disstria* last instar larvae ranges from 15 °C to 31 °C [48,95,96], the cell lines were incubated at 15, 21, 28, and 31 °C. Cell growth was assessed in situ, in terms of cells per mm^2^ using a stereo-dissecting microscope (magnification 50×). Temperature effects on cell line growth were monitored for 132 h. Subsequently, cell counting became problematic due to the formation of three-dimensional cell masses, in which hemocytes adhered both to the polystyrene substrate and to adjacent cells, thereby limiting accurate counting. Trypsin treatment failed to dissociate these cellular aggregates. The optimal growth temperature determined for each cell line was employed in all subsequent experiments.

To determine if the effects of growth at suboptimal temperatures were reversible, two of the most temperature-sensitive cell lines, UA-Md221 and UA-Md203, which exhibited little or no growth at 15 °C, were shifted from 15 °C to 21 °C, a temperature that does permit their growth, and the effects of this shift on growth parameters were determined. The recovery test was done using these temperatures since this would simulate environmental conditions experienced by fifth and sixth instar *M. disstria* larvae in the collection areas (Peace River and Edmonton) (average of maximum in Edmonton and the Peace River area for the period May 15 to June 15 recorded at 18.3 ± 5.5 °C and 17.8 ± 4.9 °C, respectively).

### 2.4. Discharge of Enzymes from Hemocyte Cell Lines in Culture Medium

Culture medium (10 mL) from flasks with adhering cell cultures at 50% confluency, grown at the optimum temperature, was collected and centrifuged (325× *g*, 4 min, 21 °C) to remove hemocyte debris. The supernatant was examined for its enzyme content.

For the continuously floating cell line Md66, enzyme content was assessed at two different times, at the time of flask inoculation and at the time when the cell culture reached 50% confluency, at their optimum temperature. In each of the two time points, samples of the cell culture were centrifuged (325× *g*, 4 min, 21 °C), and the supernatant was collected and examined for its enzyme content. Relative differences of enzyme activities between inoculation time and 50% confluency of the Md66 cell culture were used to assess the enzyme discharge in the culture medium.

The release of alkaline phosphatase and acid phosphatase, esterase (C4), esterase lipase (C8), lipase (C14), leucine aminopeptidase, valine-aminopeptidase, cystine- aminopeptidase, trypsin, chymotrypsin, acid phosphatase, phosphoamidase, α-galactosidase, β-galactosidase, β-glucuronidase, α-glucosidase, β-glucosidase, N-acetyl-β-glucosamidinase, α-mannosidase, and α-fucosidase by hemocyte cell lines was determined qualitatively and semi-quantitatively using the APIZYM test strips (BioMerieux Inc., Montreal, QC, Canada). Test strips were incubated with 65 μL volumes of culture medium, and the strips were incubated (4 h, 21 °C) and developed as directed by the manufacturer. Identification of enzymes and semi-quantitative estimation of enzyme activities were based on visual estimations of reaction-associated color development. Heat-inactivated fetal bovine serum was also examined for enzyme content. Additionally, the serum was diluted to 8% (*v*/*v*) in both PBS and Grace’s medium, adjusted to pH 6.5, and analyzed for enzyme presence.

### 2.5. Hemocyte Morphological Characterization During Growth

Cell morphogenesis, type frequencies, and blebbing behavior were assessed via phase contrast microscopy in adhering hemocyte cultures examined in situ. For analysis of floating hemocytes from the Md66 line, 80 μL aliquots of tissue culture fluid were distributed across 1 cm^2^ glass slide surfaces to facilitate cell spreading. Morphometric characterization across culture conditions encompassed determination of cell shape; maximum length or diameter; and, where applicable, documentation of appendage formation. Photographs of hemocytes were taken with a Nikon Coolpix 990 camera (Nikon Canada Inc., Mississauga, ON, Canada) on an Olympus BH2 phase contrast microscope (Olympus, Brooklyn Park, MN, USA) at 40× magnification.

Blebbing, an indicator of stress and apoptosis in hemocytes [25,26,97] and cytoplasmic release in stressed cells [98], was quantified during cell growth.

### 2.6. Discharge of Enzymes from Hemocytes Reacting to Foreign Matter in Phosphate-Buffered Saline (PBS)

Hemocyte responses to antigens may be affected by culture medium composition. Fetal bovine serum contains growth factors that affect eukaryotic cell differentiation and spreading [99], including insulin-like molecules that promote hemocyte proliferation [100] and mitotic division in granular cells of *B. mori* [101]. Additionally, integrin β1 in the serum [99] may affect hemocyte adhesion as these protein receptors participate in plasmatocyte encapsulation [29]. Medium sugars can also diminish lectin-mediated hemocyte coagulation [102] and opsonic phenoloxidase activity [103]. Thus, to elucidate the response of the hemocytes to polystyrene and dead *B. subtilis* without possible modulation by the culture medium, the hemocytes were washed free of culture medium using PBS. Md66 and Md108 were used to preclude any possible heterogeneity in non-self-responses, which could be attributed to geographic locations and/or dealing with an insect species complex. Md66 was washed by centrifugation (325× *g*, 4 min, 21 °C), and the pellet was resuspended in PBS (5 mL). Md108 was rinsed with PBS (5 mL). Hemocyte viability, based on the exclusion of the vital stain, trypan blue (0.1% *w*/*v* in PBS), was greater than 85–90%. Both Md66 and Md108 cell lines at 50% confluency (Md66, 1.4 × 10^6^ cells/mL PBS; Md108, 150 cells/mm^2^) were incubated in culture flasks without and with *B. subtilis* (1.6 × 10^8^ bacteria/mL PBS) in the buffer (5 mL) for selected periods of time (0–40 min) at 21 °C. Bacterial controls contained *B. subtilis* in PBS. Samples were centrifuged (325× *g*, 4 min, 21 °C) to remove the cell debris, and the supernatants were analyzed for enzymes.

### 2.7. Statistics

Data were analyzed using the 95% confidence limits overlap protocol, and graphic and tabular data are presented as means ± standard error of the mean [104]. Ten replicates were used unless stated otherwise. Prism 8.0 (GraphPad, Boston, MA, USA) was used for data analysis and graph presentation, while heat map clustering and principal component analysis (PCA) were performed using ClustVis (https://biit.cs.ut.ee/clustvis/ (accessed on 17 November 2025)) [105].

## 3. Results

### 3.1. Optimum Temperature for Cell Growth

**Figure 1:**
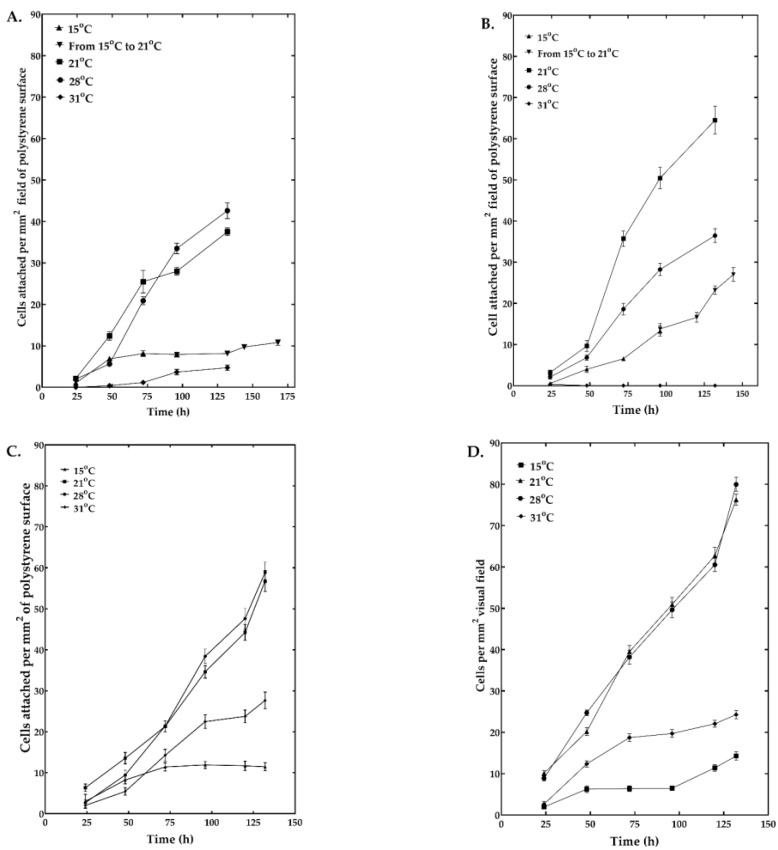
Growth of *M. disstria* hemocyte cell lines UA-Md221 (**A**), UA-Md203 (**B**), Md108 (**C**), and Md66 (**D**) on polystyrene surfaces at selected temperatures (*n* = 3 flasks, 25 random fields examined in each flask).

The optimum temperature for growth was 28 °C for all isolates except UA-Md203, for which 21 °C was optimal, and Md66, which grew best at 21–28 °C (Figure 1A,D). However, this does not mean that the cell lines exhibited similar growth profiles at their optimum or suboptimum temperatures. Although UA-Md221 cells grew marginally during the first 24 h at 15 °C, returning these cells to 21 °C at 96 h post-inoculation did not restore growth during the remaining 72 h of observation, indicating temperature-induced hemocyte damage as opposed to cytostatic effects (Figure 1A). Cell multiplication was slow but marginal at 31 °C. UA-Md203, unlike the other cell lines, grew more rapidly at 21 °C than at 28 °C, slowly at 15 °C, and not at 31 °C (Figure 1B). The UA-Md203 cell line grew more quickly upon shifting the culture from 15 °C to 21 °C.

Md108, which exhibited comparable growth profiles and rates at 21 °C and 28 °C, grew to a significantly lesser extent and slower rate at 31 °C and lesser still at 15 °C, respectively (Figure 1C). Growth patterns and rates similar to those of Md108 were seen for Md66.

The projected inoculum adhesion level was anticipated to reach 8 cells/mm^2^ by 24 h; however, the actual cell counts at 24 h post-inoculation varied according to the hemocyte cell line. Across all tested temperatures, Md108 demonstrated adhesion densities of 2–7 cells/mm^2^, while UA-Md221 exhibited 2–4 cells/mm^2^, and UA-Md203 showed 1–4 cells/mm^2^. The differences in cell adhesion are possibly due to a combination of protease effect and possible cell lysis, medium adaptation, and/or temperature effects. Similarly, variation in cell counts observed in the floating cell line may be attributable to comparable microenvironmental and physiological adaptation factors.

### 3.2. Characterization of Cell Lines in Terms of Blebbing

**Figure 2:**
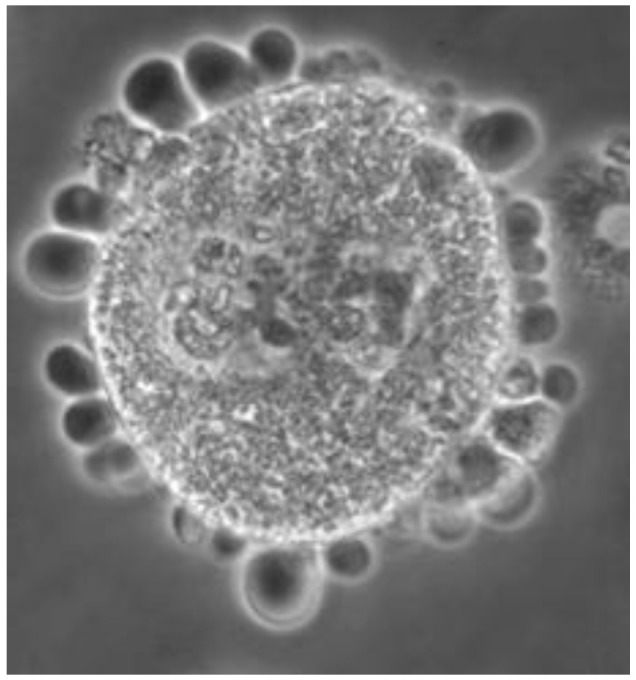
Representative caption of extensive blebbing in Md108 cell type 1 from a 40-day-old culture, at 31 °C.

**Figure 3:**
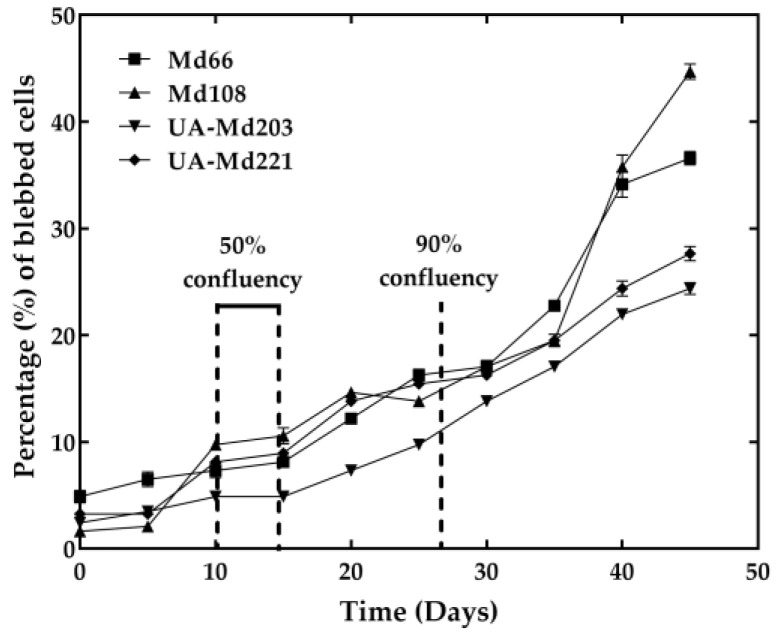
Levels of cell blebbing in Md66, UA-Md203, Md108, and UA-Md221 *M. disstria* cell lines during their incubation at optimum temperature for growth.

Cell stress in culture medium, as evidenced by blebbing (Figure 2), was minimally observed during the first 10 days of cell growth, affecting fewer than 10% of hemocytes when all cell lines remained below 50% confluency. The time required to reach 50% confluency ranged from 10 to 15 days post-inoculation, varying according to cell line. Blebbing increased concomitantly with rising confluency levels, particularly after cultures exceeded 90% confluency, typically around 30 days post-inoculation (Figure 3). Among all cell lines examined, UA-Md203 exhibited the lowest incidence of blebbing at all confluency levels, whereas the remaining cell lines demonstrated comparable blebbing frequencies between days 10 and 35 post-inoculation. Cell aggregation was observed across all cell lines during late culture stages when confluency exceeded 90%. Blebbing was not detected in any cell line during brief exposure to PBS, despite hemocytes being at 50% confluency. This observation suggests that blebbing is unlikely to have contributed to the enzyme release profiles observed in cells exposed to PBS.

### 3.3. Cell Types

**Figure 4:**
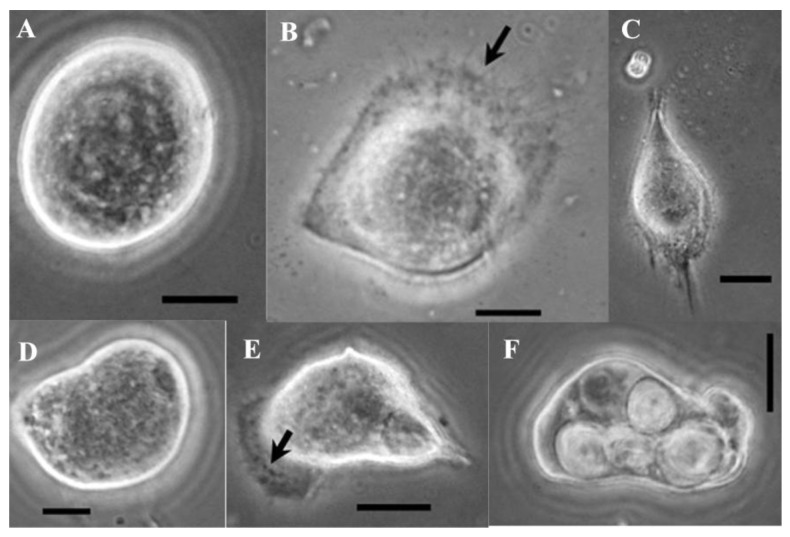
Morphogenesis of *M. disstria* Md66 hemocytic cell line: Md66 cell type 1 (**A**), Md66 cell type 2 with some membrane extension (arrow) (**B**), Md66 cell type 3 (**C**), Md66 cell type 4 (**D**), Md66 cell type 5 with crenated membrane (arrow) (**E**), and Md66 cell type 6 (**F**) (bars = 5 μm).

**Figure 5:**
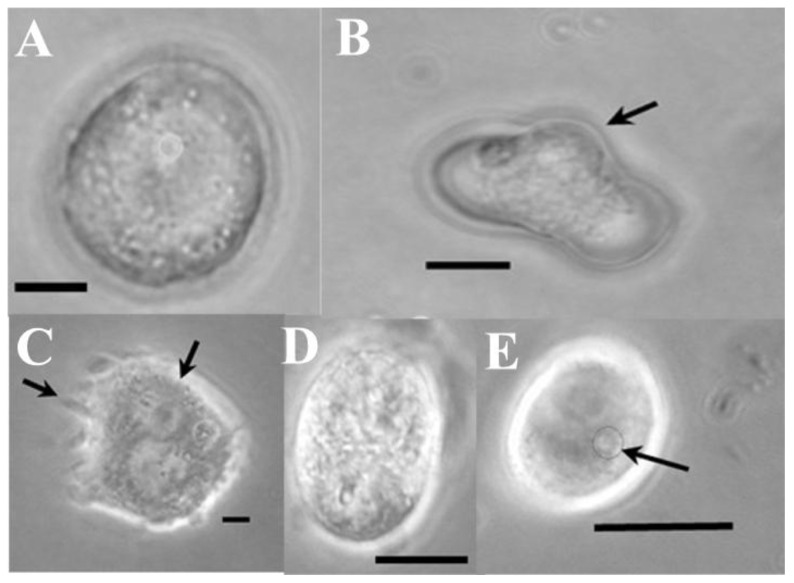
Morphogenesis of *M. disstria* Md108 hemocytic cell line: Md108 cell type 1 (**A**), Md108 cell type 2 (**B**), Md108 cell type 3 with filopodia (left arrow) extending beyond the plasmacytoid membrane (right arrow) (**C**), Md108 cell type 4 (**D**), and Md108 cell type 5 with small eccentric nucleus (arrow) (**E**) (bars = 5 μm).

**Figure 6:**
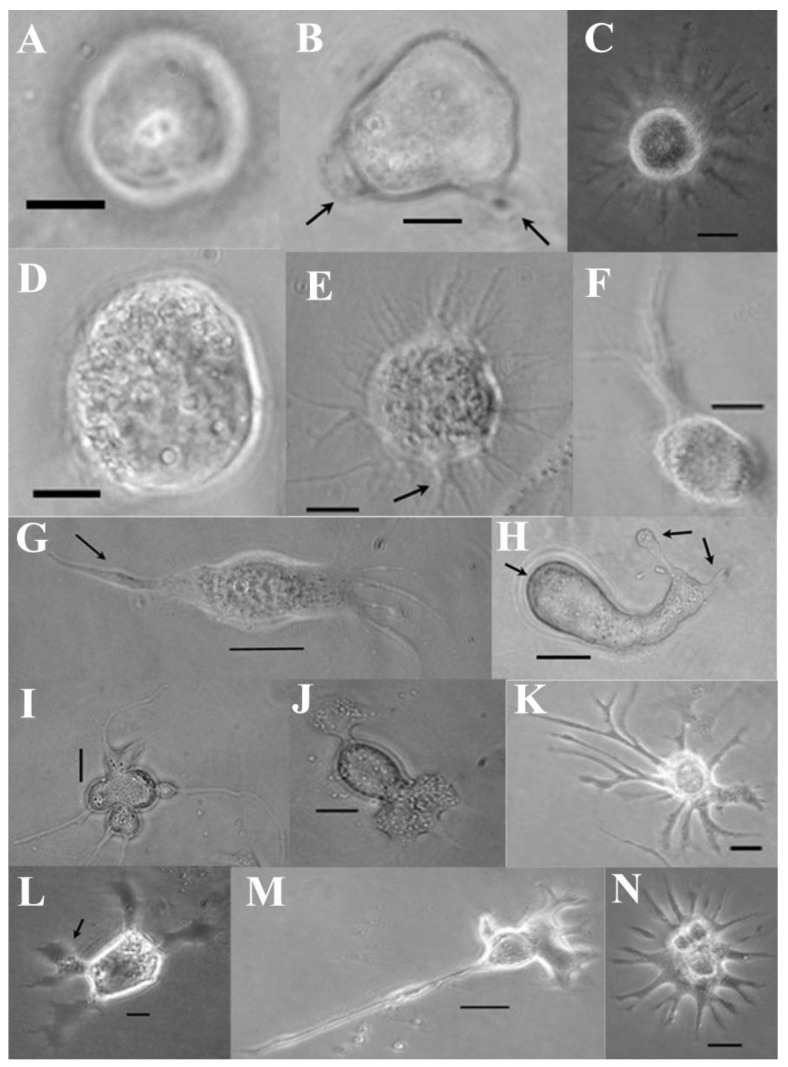
Morphogenesis of *M. disstria* UA-Md221 hemocytic cell line: UA-Md221 cell type 1 (**A**), UA-Md221 cell type 2 with lobopodial projection (arrow) (**B**), UA-Md221 cell type 3 (**C**), UA-Md221 cell type 4 (**D**), UA-Md221 cell type 5 with filopodial projections (arrow) (**E**), UA-Md221 cell type 6 (**F**), UA-Md221 cell type 7 with stylet-type projections (arrow) (**G**), UA-Md221 cell type 8 with globular projections (arrow) (**H**), UA-Md221 cell type 9 (**I**), UA-Md221 cell type 10 (**J**), UA-Md221 cell type 11 (**K**), UA-Md221 cell type 12 with enlarged lobopodia (arrow) (**L**), UA-Md221 cell type 13 (**M**), and UA-Md221 cell type 14 (**N**) (bars = 5 μm).

Cell type composition during and following subculturing varied among the UA-Md221, Md108, and Md66 cell lines, as shown in Figure 4, Figure 5 and Figure 6. Detailed descriptions of the characteristics defining each cell type category are provided in Table 1, Table 2 and Table 3. For the UA-Md221 and Md108 cell lines, recovery from stress induced by the dissociation solution—evidenced by the appearance of morphological forms absent during normal growth and detectable only following protease treatment—occurred within 48–76 h and 24–48 h, respectively.

### 3.4. Cell Frequencies

**Figure 7:**
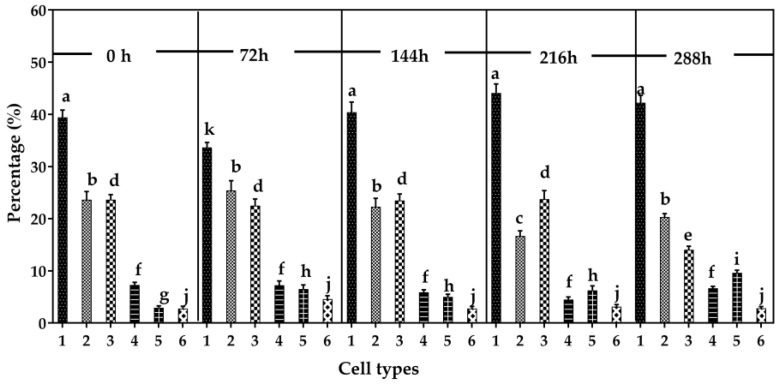
Cell type frequencies in *M. distria* hemocyte cell line Md66. Bar values with the same letter on the top are not significantly different (*n* = 10).

In Md66 cultures, cell type 1 was the most frequently observed and maintained relatively constant levels throughout the 288 h incubation period (Figure 7). Cell types 3, 4, and 6 also remained at relatively constant levels during most of the incubation period. However, the levels of cell type 2 declined at 216 h and returned to the usual level at 288 h, whereas cell type 3 declined at 288 h. Cell type 5 increased biphasically to a plateau level from 72 h to 216 h and then again by 288 h (Figure 7).

**Figure 8:**
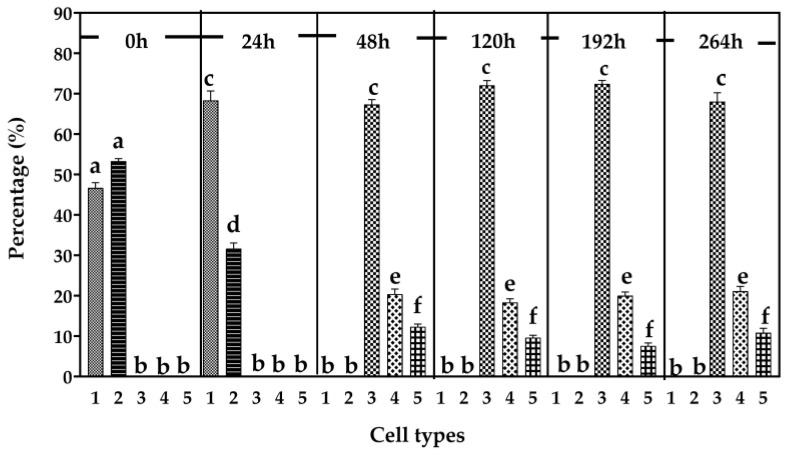
Cell type frequencies in *M. distria* hemocyte cell line Md108. Bar values with the same letter on the top are not significantly different (*n* = 10).

Trypsin treatment altered the distribution of hemocyte types in the Md108 cell line (Figure 8). Cell types 3–5 were not observed until 48 h post-trypsin exposure, by which time cell types 1 and 2 were no longer detectable. This temporal pattern suggests that Md108 cell types 1 and 2 may represent a stress response to trypsin treatment. Although the relative proportions of Md108 cell types 3–5 differed from one another, their levels remained stable from 48 h onward.

Due to the considerable morphological heterogeneity observed among hemocytes of the UA-Md221 cell line, cell type frequency analysis could not be performed at the optimum growth temperature.

### 3.5. Discharge of Enzymes from Malacosoma Disstria Cell Lines in Culture Medium

**Figure 9:**
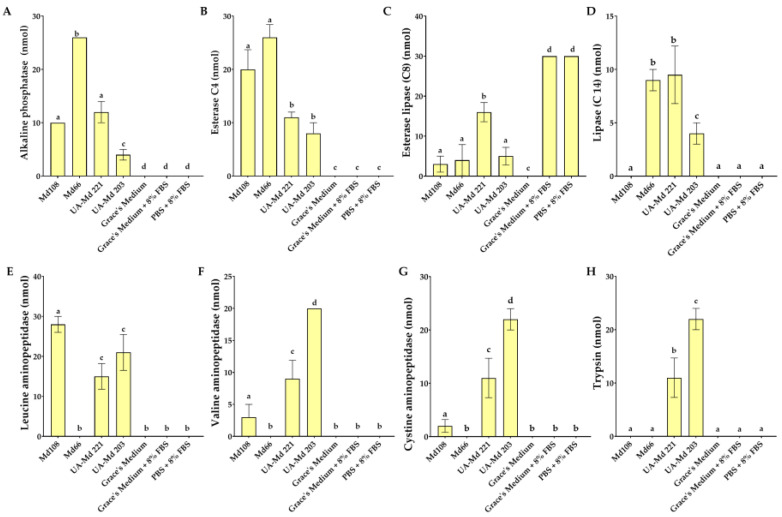
Basal levels of enzymes released from *M. disstria* hemocytic cell lines Md108, Md66, UA-Md221, and UA-Md203 at their optimum temperature to 50% confluency in culture medium [alkaline phosphatase (**A**), esterase (C4) (**B**), esterase lipase (C8) (**C**), lipase (C 14) (**D**), leucine aminopeptidase (**E**), valine aminopeptidase (**F**), cystine aminopeptidase (**G**), and trypsin (**H**)]. Enzymes are expressed in nmol substrate hydrolyzed/65 μL culture supernatant/4 h. Mean ± standard error of the mean (*n* ≥ 3). Values with the same superscript character set are not significantly different for the specific enzyme.

**Figure 10:**
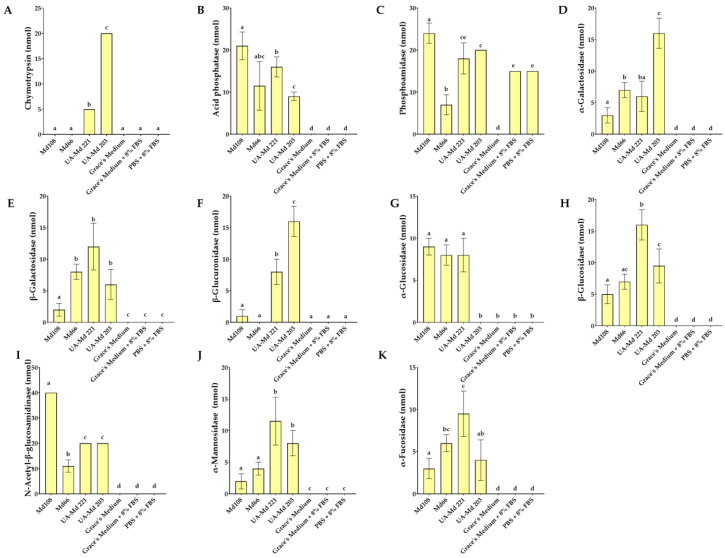
Basal levels of enzymes released from *M. disstria* hemocytic cell lines Md108, Md66, UA-Md221, and UA-Md203 at their optimum temperature to 50% confluency in culture medium [chymotrypsin (**A**), acid phosphatase (**B**), phosphoamidase (**C**), α-galactosidase (**D**), β-galactosidase (**E**), β-glucuronidase (**F**), α-glucosidase (**G**), β-glucosidase (**H**), N-acetyl-β-glucosamidinase (**I**), α-mannosidase (**J**), and α-fucosidase (**K**)]. Enzymes are expressed in nmol substrate hydrolyzed/65 μL culture supernatant/4 h. Mean ± standard error of the mean (*n* ≥ 3). Values with the same superscript character set are not significantly different for the specific enzyme.

Analysis of centrifuged spent media revealed that UA-Md221 and UA-Md203 cultures produced comparable levels of esterase lipase (C8), phosphoamidase, and N-acetyl-β-glucosaminidase. However, the supernatant from UA-Md221 cultures exhibited higher acid phosphatase activity, whereas UA-Md203 cells released greater amounts of valine-aminopeptidase, cystine-aminopeptidase, α-galactosidase, and β-glucuronidase (Figure 9F,G and Figure 10D,F). Both adherent cell lines released the serine proteases trypsin and chymotrypsin, with UA-Md203 spent medium demonstrating higher activity levels of both enzymes compared with UA-Md221.

Spent supernatants from adherent Md108 cells contained significantly lower esterase (C4) activity compared with those from UA-Md221 and UA-Md203 culture (Figure 9B). Esterase lipase (C8) levels were highest in UA-Md221 supernatants, whereas the other two adherent cell lines exhibited comparable enzyme activity (Figure 9C). The moderately adherent cell lines Md108 and UA-Md203 released higher levels and a greater diversity of enzymes compared with UA-Md221. Specifically, Md108 secreted elevated levels of esterase (C4), leucine aminopeptidase, and N-acetyl-β-glucosaminidase relative to both UA-Md221 and UA-Md203. In contrast, the culture supernatant from UA-Md203 contained higher concentrations of valine aminopeptidase, cystine aminopeptidase, trypsin, and chymotrypsin than supernatants from the other adherent cell lines (Figure 9F–H and Figure 10A). No discernible relationship was observed between trypsin and chymotrypsin activity and hemocyte adhesion avidity among the three adherent cell lines.

α-mannosidase (Figure 10J) and both acid and alkaline phosphatases (Figure 9A and Figure 10B) were released into the culture medium by all hemocyte cell line types. Elevated α-mannosidase activity was observed in media from UA-Md221 and UA-Md203 cell lines, whereas Md108, which released the lowest levels of α-mannosidase, exhibited the highest secretion of both phosphatase types.

The Md66 cell line released fewer enzyme types into the culture medium compared with the other cell lines examined. No evidence of trypsin or chymotrypsin release was detected (Figure 9H and Figure 10A). Owing to the subculturing protocol for Md66, in which the inoculum transferred to fresh medium contained enzymes from the preceding culture, direct comparisons of medium enzyme content with adherent cell lines were not feasible.

Fetal bovine serum diluted with PBS or culture medium revealed detectable amounts of esterase lipase (C8) and phosphoamidase activities (Figure 9C and Figure 10C). Incubation of cell lines in medium supplemented with fetal bovine serum resulted in decreased esterase lipase activity, whereas adherent cell lines demonstrated elevated phosphoamidase levels.

### 3.6. Comparative Analysis of Cell Line Enzyme Release in Culture Medium

The analysis of enzyme activity patterns across different hemocytic cell lines and culture conditions reveals distinct metabolic signatures that clearly separate these samples into meaningful groups. Τhe four cell lines (Md108, Md66, UA-Md 221, and UA-Md 203) show clear metabolic differences from one another. The UA-Md cell lines (e.g., 221 and 203) demonstrate particularly distinctive enzyme profiles that set them apart from Md108 and Md66, suggesting these represent different metabolic phenotypes or adaptation strategies. The non-inoculated culture media and buffer (Grace’s Medium alone, Grace’s Medium with 8% FBS, and PBS with 8% FBS) form a separate cluster entirely, showing minimal enzyme activity across most measured parameters. Interestingly, the addition of fetal bovine serum (FBS) to either Grace’s Medium or PBS produces nearly identical enzyme qualitative and quantitative profiles. Cell lines express highly variated (a) phosphatase activities (both alkaline and acid phosphatases), (b) esterase enzymes, and (c) specific glycosidases (α-glucosidase and α-galactosidase), suggesting different metabolic specializations among the cell lines and nutrient source adaptations on their optimal thermal exposure conditions.

**Figure 11:**
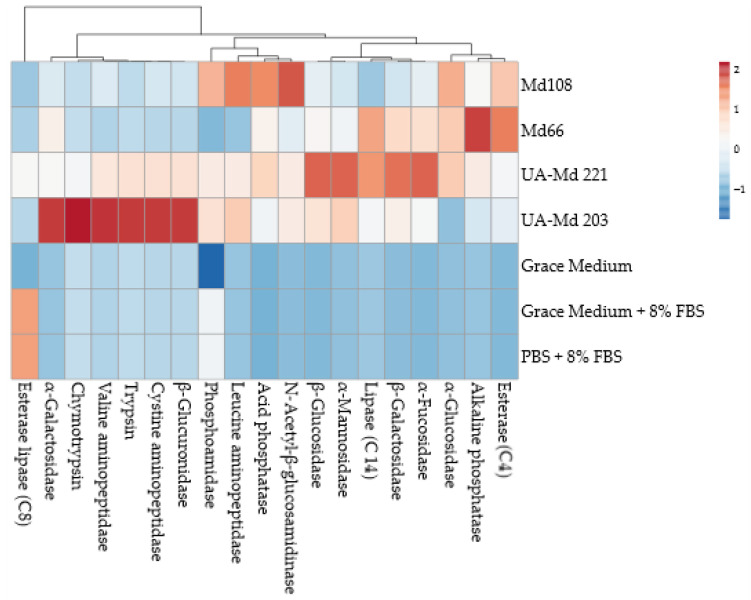
Heatmap of enzyme release of *M. disstria* hemocytic cell lines Md108, Md66, UA-Md221, and UA-Md203 in culture medium at optimal temperature for each one and at 50% confluency. The variability in enzyme release from cells to medium decreased in the following order: Md203 > Md221 > Md108 ≈ Md66.

Several enzymes show particularly interesting patterns; for example, aminopeptidases (leucine, valine, and cystine) show strong activity in UA-Md203 and UA-Md221 cell lines but are largely absent in Md108 and Md66, and glycosidase enzymes display complex patterns, which help distinguish between all four of them. The hemocytic cell lines exhibit rich enzymatic profiles suggesting active metabolism and diverse substrate utilization capabilities. The UA-Md203 and UA-Md221 cell lines appear to apply extensive protein degradation (aminopeptidases) and complex carbohydrate processing. In a different physiological route, the Md108 and Md66 cell lines show their own distinctive patterns, with Md66 displaying higher esterase (C4) and phosphatase activities compared with Md108 (Figure 11).

### 3.7. Discharge of Enzymes from Floating Md66 Hemocytic Cell Line in Response to Foreign Matter

**Figure 12:**
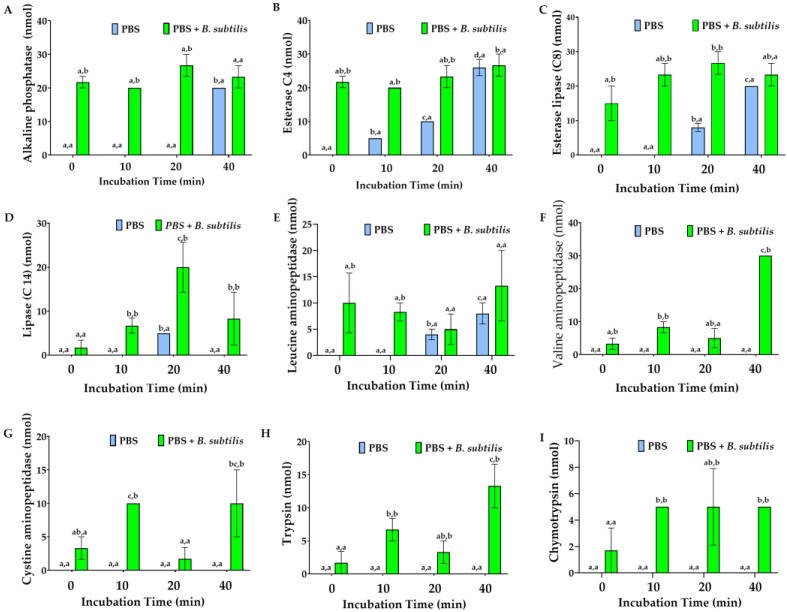
Enzymes released from Md66 hemocyte cell line into phosphate-buffered saline (PBS) in the presence and absence of *B. subtilis*: alkaline phosphatase (**A**), esterase (C4) (**B**), esterase lipase (C8) (**C**), lipase (C14) (**D**), leucine aminopeptidase (**E**), valine aminopeptidase (**F**), cystine aminopeptidase (**G**), trypsin (**H**), and chymotrypsin (**I**). Enzymes are expressed in nmol substrate hydrolyzed/65 μL culture supernatant/4 h. The hemocyte cell line is grown to 50% confluency at their optimum temperature prior to washing by centrifugation and resuspension in PBS. Values represent mean ± standard error of the mean, where (a) the same first superscript character set are not significantly different for the specific enzyme at different times and (b) the same second superscript character set are not significantly different for the specific enzyme and the specific assessment time between the two different treatments (PBS, PBS + *B. subtilis*) (*n* = 3).

**Figure 13:**
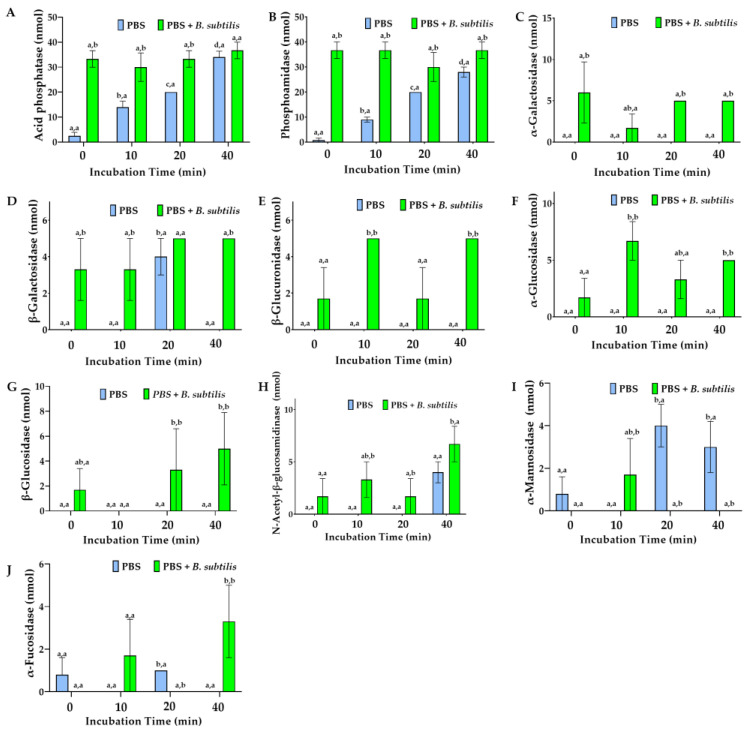
Enzymes released from Md66 hemocyte cell line into phosphate-buffered saline (PBS) in the presence and absence of *B. subtilis*: acid phosphatase (**A**), phosphoamidase (**B**), α-galactosidase (**C**), β-galactosidase (**D**), β-glucuronidase (**E**), α-glucosidase (**F**), β-glucosidase (**G**), N-acetyl-β-glucosamidinase (**H**), α-mannosidase (**I**), and α-fucosidase (**J**). Enzymes are expressed in nmol substrate hydrolyzed/65 μL culture supernatant/4 h. The hemocyte cell line is grown to 50% confluency at their optimum temperature prior to washing by centrifugation and resuspension in PBS. Values represent mean ± standard error of the mean, where (a) the same first superscript character set are not significantly different for the specific enzyme at different times and (b) the same second superscript character set are not significantly different for the specific enzyme and the specific assessment time between the two different treatments (PBS, PBS + *B. subtilis*) (*n* = 3).

Enzyme type and activity released from the hemocytes in PBS varied with the hemocyte cell line, incubation time, and antigenic stimulus (Figure 12 and Figure 13). Md66 in PBS without bacteria continuously increased esterase (C4), acid phosphatase, and phosphoamidase levels and elevated esterase lipase (C8) from 10 min to 40 min post-inoculation (Figure 12B,C and Figure 13A,B). Alkaline phosphatase increased from 20 min post-incubation time; the other enzymes either were absent or exhibited spurious activities (Figure 12A). In the presence of *B. subtilis* the number of enzymes detected at 0 min post-incubation increased from 4 in bacterial-free PBS to 17 enzymes of which consistently high activities occurred for acid and alkaline phosphatase, phosphoamidase, esterase (C4), and esterase lipase (C8) (Figure 12A–C and Figure 13A,B). Comparable activity values for these enzymes occurred at 20 or 40 min post-inoculation for Md66 cells loosely contacting the polysterene surface of the tissue culture flasks. Valine- and cystine-aminopeptidases, although absent in bacteria-free PBS with hemocytes, were detected at equal activity levels during 0–20 min incubation with *B. subtilis*, the former aminopeptidase substantially increasing thereafter by 40 min post-inoculation (Figure 12F,G). Leucine aminopeptidase consistently exhibited higher activity in the presence of bacteria than in the absence of bacteria during the time interval of 0–10 min and comparable levels thereafter. Trypsin and chymotrypsin were present in cell lines with *B. subtilis* but not in the bacteria-free cultures (Figure 12H,I). Carbohydrases occurred from 0 to 40 min post-inoculation with bacteria, their profiles differing from bacteria free-samples (Figure 13).

#### Significance of Enzyme Discharge for Md66 Cell Line Challenged with *B. Subtilis*

At the immediate level of cell line responses (0–10 min), the most important finding was the immediate enzymatic response to the presence of *B. subtilis*. The Mdd66 cell line exposed to *B. subtilis* displayed immediate activation across multiple enzyme systems, particularly in alkaline phosphatase and esterase enzymes, showing rapid activation. Esterase lipase (C8) demonstrated a consistent early response and initial activation of proteolytic enzymes at extremely low concentrations.

During the time interval of 10–20 min, control PBS samples began showing some spontaneous enzyme activity, possibly due to physicochemical and environmental factors or autolysis. However, the *B. subtilis*-treated Md66 cells consistently maintained higher activity levels and showed more organized patterns of enzyme expression, representing a critical transition point where treatment effects became most pronounced for the examined enzymes.

By 40 min, *B. subtilis*-treated Md66 cells showed the strongest deviation from all other samples, indicating a fully developed enzymatic response. Enzymes like valine aminopeptidase showed dramatic increases, suggesting specific metabolic and innate immunity pathways.

Primary response enzymes, showing immune and metabolic activation, include proteolytic enzymes (trypsin and chymotrypsin), cell wall-degrading enzymes (β-glucuronidase), aminopeptidases, and phosphatases. The enzymes that highlighted the contrast between early and late responses were α-fucosidase, acid phosphatase, and esterase (C4), which showed early activation, and α-mannosidase, which displayed unique temporal patterns distinct from other glycosidases.

**Figure 14:**
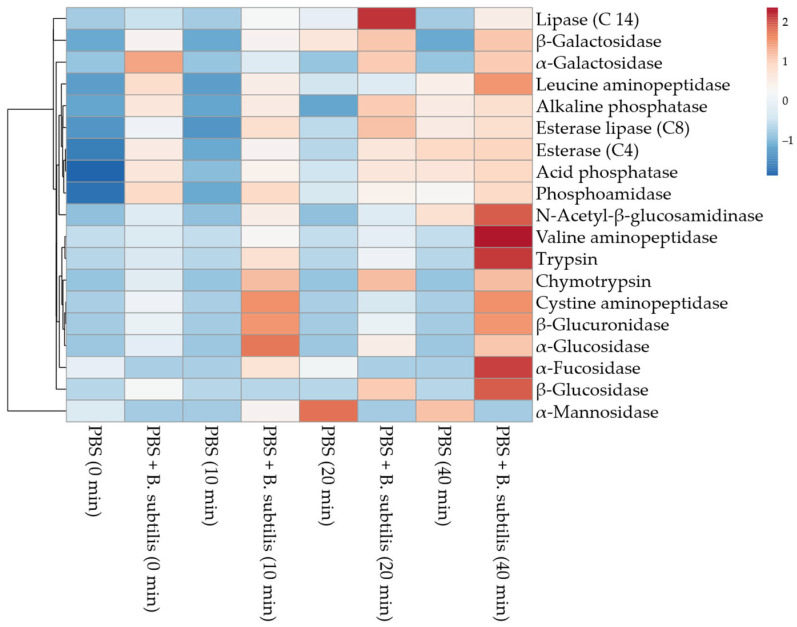
Heatmap of enzyme release of *M. disstria* hemocytic cell line Md66 challenged with *B. subtilis* in PBS.

*B. subtilis* triggered innate and metabolic responses in Md66 cells. The interaction was extremely complex, but it underlies a hierarchy in enzyme activities: immediate activation of phosphatases and esterases, which represent the initial topological cellular signaling on the Md66 cell membrane, followed by progressive activation, where protein-processing enzymes act, and finally complex carbohydrate-degrading enzymes for metabolic or immunological adaptation. Clear temporal patterns emerge of potential biomarkers for the early detection of bacterial presence (phosphatases and esterases), monitoring the progression of host–microbe interactions (aminopeptidases), and metabolic status assessment (glycosidases) (Figure 14).

### 3.8. Discharge of Enzymes from Adherent Md108 Hemocytic Cell Line in Response to Foreign Matter

**Figure 15:**
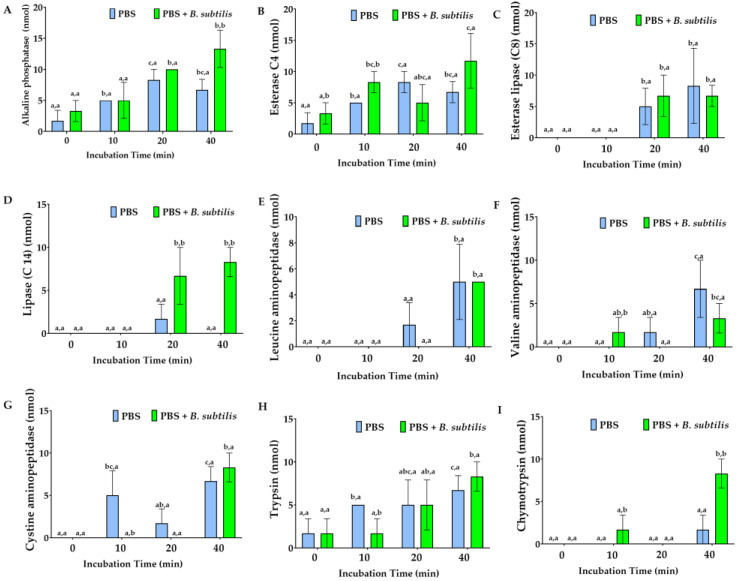
Enzymes released from *M. disstria* Md108 hemocyte cell line into phosphate-buffered saline (PBS) in the presence and absence of *B. subtilis*: alkaline phosphatase (**A**), esterase (C4) (**B**), esterase lipase (C8) (**C**), lipase (C14) (**D**), leucine aminopeptidase (**E**), valine aminopeptidase (**F**), cystine aminopeptidase (**G**), trypsin (**H**), and chymotrypsin (**I**). Enzymes are expressed in nmols substrate hydrolyzed/65 μL culture supernatant/4 h. The hemocyte cell line is grown to 50% confluency at their optimum temperature prior to washing by centrifugation and resuspension in PBS. Values represent mean ± standard error of the mean, where (a) the same first superscript character set are not significantly different for the specific enzyme at different times and (b) the same second superscript character set are not significantly different for the specific enzyme and the specific assessment time between the two different treatments (PBS, PBS + *B. subtilis*) (*n* = 3).

**Figure 16:**
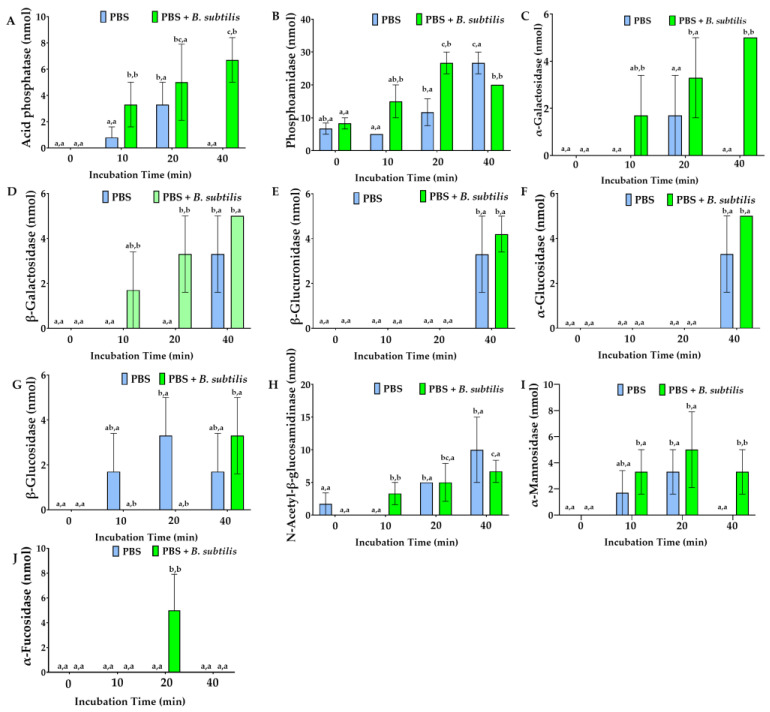
Enzymes released from *M. disstria* Md108 hemocyte cell line into phosphate-buffered saline (PBS) in the presence and absence of *B. subtilis*: acid phosphatase (**A**), phosphoamidase (**B**), α-galactosidase (**C**), β-galactosidase (**D**), β-glucuronidase (**E**), α-glucosidase (**F**), β-glucosidase (**G**), N-acetyl-β-glucosamidinase (**H**), α-mannosidase (**I**), and α-fucosidase (**J**). Enzymes are expressed in nmols substrate hydrolyzed/65 μL culture supernatant/4 h. The hemocyte cell line is grown to 50% confluency at their optimum temperature prior to washing by centrifugation and resuspension in PBS. Values represent mean ± standard error of the mean, where (a) the same first superscript character set are not significantly different for the specific enzyme at different times and (b) the same second superscript character set are not significantly different for the specific enzyme and the specific assessment time between the two different treatments (PBS, PBS + *B. subtilis*) (*n* = 3).

*B. subtilis* treatment on the Md108 cell line revealed time-dependent enzymatic enhancement, with peak activities at 40 min post-treatment (Figure 15 and Figure 16). Phosphatases (alkaline and acid) showed pronounced upregulation, indicating altered phosphate metabolism and cellular signaling. Aminopeptidases, particularly leucine, valine, and cystine variants, increased substantially, suggesting enhanced protein turnover and amino acid liberation. Carbohydrate-active enzymes exhibited marked elevation at later timepoints, facilitating polysaccharide degradation and carbohydrate utilization. The temporal progression revealed minimal changes at 10 min, moderate increases at 20 min, and maximal responses at 40 min across all enzyme classes.

#### Significance of Enzyme Discharge for Md108 Cell Line Challenged with *B. Subtilis*

**Figure 17:**
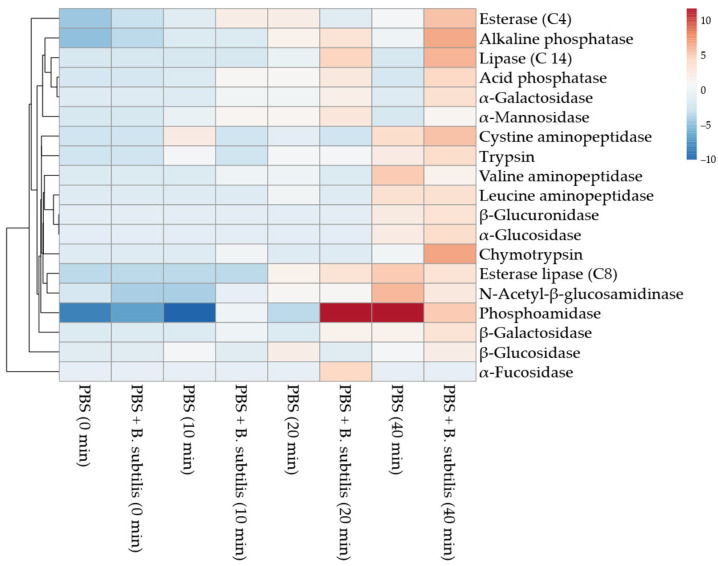
Heatmap of enzyme release of *M. disstria* hemocytic cell line Md108 challenged with *B. subtilis* in PBS.

Clustering analysis of Md108 cell line responses upon *B. subtilis* challenge demonstrated that enzymes operate within interconnected networks, responding collectively in distinct groups (Figure 17). The immediate response group, consisting of esterase (C4) and alkaline phosphatase, forms a tightly linked pair that reacts upon bacterial detection by Md108 hemocytes. This synchronized response indicates their role in the initial recognition and/or membrane interactions caused by the presence of microbes. Thereafter, the proteolytic enzyme group became predominant in the interaction being studied. Aminopeptidases (cystine, valine, and leucine) clustered together, while trypsin and chymotrypsin exhibited similar activation patterns. This proteolytic cluster suggests a coordinated effort in protein degradation and processing for both metabolic and immunological functions. Additionally, the carbohydrate-processing enzyme group, which includes glycosidases (α and β), β-galactosidase, and β-glucosidase, shows correlated activity in the breakdown of complex carbohydrates. Interestingly, α-mannosidase displayed an inverse response pattern, decreasing as the others increased, while lipase (C14) showed a delayed and moderate activation. Acid phosphatase acted as a bridge between the early and late response groups. Observations made within the 0–10 min time interval were most effective for detecting initial contact and early responses, whereas the 20–40 min period was optimal for observing response transitions and the diversification of immune and metabolic profiles.

### 3.9. Comparison of Md66 and Md108 Cell Lines Enzyme Discharge in Presence of B. subtilis

Principal component analysis revealed that physiological subtype accounted for 58% of the enzyme activity variation, reflecting distinct metabolic and immunological responses across cell lines. When correlated with time-dependent enzymatic release patterns, this inter-line variation increased to 73%. The Md66 cell line exhibited metabolic and immune readiness through immediate high enzyme activity and nonlinear release dynamics. Valine aminopeptidase activity peaked sharply at 40 min post-bacterial exposure, while multiple enzyme classes—phosphatases, esterases, peptidases, and glycosidases—showed broad temporal release patterns.

Md108 exhibited a distinct gradual activation pattern, initiating with near-zero enzyme activity except for trace levels of alkaline phosphatase and esterase (C4) (Figure 15). This suggests either delayed gene expression or regulatory control under specific conditions. The most significant changes occurred between 20 and 40 min, suggesting the necessity of prolonged exposure to achieve complete activation and narrower spectrum of enzyme release, in contrast to Md66. Unlike Md66, Md108 demonstrated a narrower enzymatic spectrum, with certain enzymes such as leucine aminopeptidase maintaining consistently low activity levels throughout the observation period.

Md108 exhibited partially overlapping enzyme release patterns, with alkaline phosphatase remaining consistently elevated in Md66 while gradually increasing in Md108. Both cell lines showed enhanced trypsin and chymotrypsin release at 40 min post-bacterial challenge.

Alkaline phosphatase and esterase (C4) exhibited similar trends with distinct kinetics, while esterase lipase (C8), lipase (C14), and leucine aminopeptidase displayed cell line-specific patterns. α-mannosidase showed contrasting responses between the two cell lines. Both N-acetyl-β-glucosamidinase and α-fucosidase exhibited temporal patterns specific to each cell line, with the latter enzyme displaying significantly different release timings.

**Figure 18:**
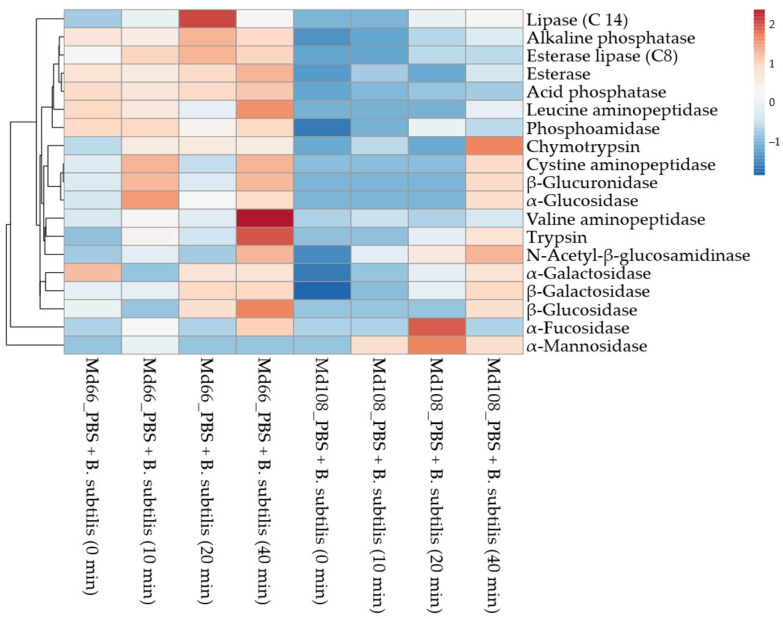
Comparative heatmap of *M. disstria* Md66 and Md108 cell lines enzyme release challenged with *B. subtilis* in PBS.

During the first 10 min of bacterial exposure, the cell lines displayed marked enzymatic differentiation that subsequently attenuated between 20 and 40 min. Md66 and Md108 consistently exhibited distinct temporal enzyme profiles and unique bacterial response patterns. Md108 exhibits a regulated response strategy, potentially driven by either signal-dependent enzyme release during bacterial challenge, resulting in delayed response kinetics, or a low-energy functional profile absent in Md66 (Figure 18).

## 4. Discussion

The growth of cell lines exhibited sensitivity to temperature variations, with each line displaying distinct thermal optimum and differential sensitivity to suboptimal and superoptimal conditions. Despite variations in temperature sensitivity, hemocytic lines consistently demonstrated poor growth at suboptimal temperatures. Herein, adhesion was temperature independent. The hemocyte responses of dipteran encapsulation of parasitoids [106] and Lepidoptera larval nodulation of bacteria [107] and hemocytic erythrocyte phagocytosis [108] diminished with decreasing temperature. This may explain, in part, why *M. disstria* larvae, which grow slowly at low temperatures [109], exhibit an increase in mycoses when exposed to the entomopathogenic fungus *Furia gastropachae* (Zygomycetes: Entomophthorales) [110], the hemocytes possibly having diminished activity (present study) even as the temperature enhances the pathogen [110,111].

UA-Md203 exhibited an optimal growth temperature of 21 °C, which was distinct from that of other cell lines (21–28 °C). At 15 °C, both UA-Md203 and UA-Md221 showed reduced growth with line-specific responses. UA-Md203 maintained continuous but slow growth at 15 °C, with increased proliferation upon shifting to 21 °C, suggesting a cytostatic effect. However, its growth remained slower than that of cells continuously cultured at 21 °C, indicating either chronic damage or selection for cold-adapted cell subtypes. In contrast, UA-Md221 displayed initial growth at 15 °C, followed by complete growth arrest that persisted after the temperature shift to 21 °C, demonstrating a cytocidal effect. The accelerated growth rates of UA-Md221 and UAMd203 compared with previous reports are likely due to differences in fetal bovine serum concentration (6% *v*/*v*; 8% *v*/*v* herein) and/or compositional variations [56]. The limited growth of Md66 and Md108 at 15 °C may reflect hemocyte properties linked to the initial Md66 selection from an adherent cell line, potentially influenced by the distinct geographical origins of the larval source [92].

The temperature response differences that were observed in hemocytes during these studies may reflect the possibility of *M. disstria* being a species complex [112] that is morphologically difficult to separate, a phenomenon that occurs also in other insect species [113]. The nucleotide sequences of the mitochondrial gene from *M. disstria* cytochrome oxidase I from different locations support this contention [112]. It is unlikely that the differences represent selection by cultivation temperature because the lines were all grown at similar temperatures, 25–28 °C [56,59,114]. Growth at 31 °C was highly variable among the cell lines with lowest rates in UA-Md203 and highest at Md108. The limited to no growth and slow growth may reflect medium degeneration as determined for a *G. mellonella* ovarian cell line [115] and a larval fat body cell line [116].

Hemocyte cell lines exhibited a greater degree of morphological diversity compared with freshly isolated hemolymph samples from *M. disstria* larvae [39]. Charpentier et al. [117] refer to the polyploidy state of insect hemocyte cell lines of *Leptinotarsa decemlineata* as a possible factor in cellular polymorphism. RNAi studies in *D. melanogaster* embryonic hemocyte lines identified 994 cell shape regulators, which could potentially explain the observed spectrum of hemocyte phenotypes [118]. Despite the morphological differences between the cell lines and the freshly isolated hemocytes [39], serological properties of Md66 cell line show similarities with *M. disstria* larvae hemolymph samples, which imply that Md66 floating hemocytes demonstrate similar properties to fresh blood samples [119]. Herein, the UA-Md221 cell line exhibited extensive polymorphism. Such polymorphism has not been shown in direct blood sampling from the larval hemolymph [39] possibly due to the absence of plasma factors in the cell lines. The contribution of protease treatments for one hour of UA-Md221 cells and their cell shape pattern stability while proliferating should be further investigated since trypsin alters animal cell surface glycoproteins and their in situ regenerative synthesis, which could have consequences on adhesion shape pattern and therefore on the overall cell shape appearance [120].

One of the major components of insect tissue culture medium, fetal bovine serum, contains animal growth factors [99] and enzymes [121] that might play a role in cell development and function. TGF-β (transforming growth factor-β) found in fetal bovine serum [99] affects molluscan immunocyte cell shape [122]; induces chemotactic, calcium dependent migration of immunocytes [122]; and upregulates epinephrine release from them [123]. The growth factor also prevents apoptosis in fat body of cell line of lepidopteran *Lymantria dispar* [124,125]. Inoculation of hemolymph samples from the tick, *Ornithodoros moubata*, with fetal bovine serum increases the phagocytic activity of the granulocytes but not that of the plasmatocytes [126]. Additionally, serum proteins generally bind rapidly to cells and their physical supports [127], the consequence for animal cells varying with the type of physical support; for example, polystyrene–serum interaction substantially affects rat glioma cell adhesion and cellular functions [128]. Phosphoamidase and esterase lipase (C8) are the two enzymes that are consistently present in fetal bovine serum whether diluted with Grace’s medium or PBS. Phosphoamidase, while it is frequently described in insect tissue culture [129,130], is commonly released by insect pathogens [131] and may participate in breakdown in the extracellular matrix proteins [132]. However, cell adhesion to flasks suggests limited matrix protein hydrolysis by this enzyme as extensive hydrolysis would inhibit hemocyte attachment. It is also present in the gut of predatory pentatomids [133] and saliva of parasitic arthropods [134] assisting protein digestion. It is not known if this enzyme digests proteins in the culture medium. Strong phosphoamidase and weak esterase lipase activities were detected during the growth of NIAS-MaBr-92 and NIAS-MaBr-93 hemocyte cell lines of the cabbage army moth, *Mamestra brassicae*, while the cells grew [130]. Esterase-lipase (C8) was detected in the venom of the wasp *Pimpla hypochondriaca*, hymenopteran parasitoid of bright-line brown-eye moth, *Lecanobia oleracea* larvae; however, the exact contribution of the enzyme to the immunosuppresive venom antihemocytic activity or antimicrobial spectrum of the parasitoid is not known [135]. Antimicrobial properties of esterase lipase have been demonstrated in mollusk hemocytes against bacteria [136]. There is no discernible link between esterase lipase and adhesion of *M. disstria* hemocytes to polystyrene flasks since all cell lines, independently of their adhesion properties, produced these enzymes in the tissue culture medium.

Keddie et al. [56] propose that the most avidly adhering cell line, UA-Md221, may inactivate external proteases, which may herein explain the low levels of trypsin and chymotrypsin in the culture medium compared with lesser adhesive UA-Md203. It is possible also that the higher enzyme activities in UA-Md203 cultures are due to their greater production and/or release, which overcomes the documented presence of serine protease inhibitors in fetal bovine serum [137].

There was no obvious relationship between hemocyte adhesion avidity to polystyrene and serine protease activity in the culture medium, which, while possibly reflecting the aforementioned reasons for UA-Md221 and UA-Md203 cell lines, may be made more complex by the myriad of molecules found in FBS. Both mildly adhering Md108 and the floating Md66 cell line did not produce trypsin and chymotrypsin in the culture medium compared with UA-203 and UA-221. The absence of trypsin and chymotrypsin in secretion enzyme profile Md108 and Md66 could result from inhibition by the fetal bovine serum serine proteases inhibitors, e.g., an inter-α-trypsin inhibitor [137,138]. Differences in trypsin and chymotrypsin activities by Md108 and Md66 cells when suspended in tissue culture medium and PBS could have occurred because of culture conditions and absence of serine protease inhibitors from the fetal bovine serum [137] while incubated in PBS.

Although the objective of documenting activities of serine proteases and other enzymes was to establish a baseline for hemocytes under optimum temperature to ensure culture quality, examination of possible enzyme functions is also useful. Many of the enzymes showing increased activities in the culture medium are lysosomal enzymes with known antimicrobial activity [136,139,140] in mollusks [136,139] and insects [140]. Many of these enzymes are released from the hemocytes as part of the non-self-activities [84,141].

Aminopeptidases [142] are enzymes that can degrade bioactive peptides [by removing amino acids sequentially from the unblocked N termini] [143] and can limit tumor necrosis factor–alpha (TNF-α)-induced apoptosis in human neutrophils [144]. In terms of non-self-responses, molluscan aminopeptidases exhibit statistically higher activity in the circulating hemocytes and in serum of the gastropod mollusk *Biomphalaria glabrata* during infections with the parasitic trematode *Echinostoma lindoense* in respect to non-treated controls [136]. Aminopeptidases may not be linked to the inhibition of hemocyte adhesion in the present study, because although the enzymes were absent in the floating Md66 cell line, they are present in high amounts in more adhesive cell lines. Similarly, they may not enhance adhesion, since there was no discernible link between enzyme levels and hemocyte adhesion avidity. Leucine aminopeptidase is highly expressed in host immune self-cellular responses, e.g., like endometrial carcinogenesis via adipocytes [145] and immune non-self-responses as, e.g., in virally immunodeficient T-cell lymphocyte cell lines [146]. The secretion enzyme profile of human blood cells after exposure to type I interferon enhances leucine aminopeptidase activity within the first 12 h [147], which indicates this enzyme activity increases under immunological stimulation. Interferon response elements are found in the promoter region of the peptidoglycan recognition protein (PGRP) gene in Lepidoptera [148], which may imply that the release of leucine aminopeptidase by hemocytes is linked with insect cellular non-self-responses against microbial surfaces.

Secretion of N-acetyl-β-glucosaminidase occurs in the hypopharyngeal gland in eusocial bees, *Scaptotrigona postica*, and newly emerged individuals of the honeybee, *Apis mellifera* [149], which may facilitate food digestion and/or protection from the microbial content in the alimentary canal. Bovine neutrophils in culture plates releases more N-acetyl-β-glucosaminidase when leukocyte Fc receptor is stimulated than in β-integrin-deficient neutrophils; this enzyme is participating in non-self-responses [150]. Herein, there is no obvious relationship between adhesive ability and N-acetyl-β-glucosaminidase levels. Interestingly, the low levels of secretion of N-acetyl-β-glucosaminidase by hemocytes in PBS with or without bacteria do not indicate that the enzyme supports antimicrobial activity in the hemocyte cell lines.

Although β-galactosidase was present in growth medium from all hemocyte cultures at 50% confluency, enzyme activity was very low when the hemocyte cell lines were in PBS with polystyrene and bacteria. According to Tururen [151], β-galactosidase contributes to insect hemolymph by hydrolyzing glycolipids and subsequently enhancing the levels of trehalose [149], which is the major hemolymph disaccharide. Although the sugar is a target for hydrolysis by the entomopathogenic fungus *Metarhizium anisopliae* as it proliferates in the hemolymph [152], in healthy insects the non-immune modifying sugar [153] may protect the integrity of hemocytes against oxidative stress [154]. Alternatively, since insect hemolymph contains substrates for this enzyme and others considered in this discussion, it is possible those enzymes without a link to hemocyte adhesion and with or without antimicrobial potential may contribute to the hemocytes acquiring nutrients from hemolymph and herein culture medium. In *Drosophila melanogaster* Fc clone of the Kc cell line, it was demonstrated that the β-galactosidase gene is highly induced in presence of ecdysterone [155], a hormone that activates attachment mechanisms in the floating Md66 cell line [52]. The relationship between this enzyme and ecdysteroids for Md66 was not addressed, but in view of antigen–hemocyte attachment and hormone mediation of antimicrobial innate humoral [156] and hemocyte activity [157], β-galactosidase may have a non-self-function.

β-glucuronidase, an enzyme observed in hemocytes of *Galleria mellonella* with a possible antimicrobial role [158], herein was not linked to antimicrobial responses of Md66 and Md108 cell lines against *B. subtilis* or polystyrene–hemocyte reactions in PBS. The enzyme does have an antimicrobial or general non-self-role in invertebrates and vertebrates. Snail *B. glabrata* β-glucuronidase in plasma plays a role in host immunity by increasing its activity during infection by *Schistosoma mansoni* [159,160]. Distribution of β-glucuronidase in the oviduct of the domestic hen, *Gallus domesticus*, provides an antibacterial protective mechanism [161].

A positive correlation between adhesion avidity to polystyrene and α-mannosidase released in tissue culture medium among *M. disstria* cell lines is observed in the present work. The enzyme has protective roles in other animal systems. α-mannosidase may serve a protective role on the cytoplasmic membranes of *D. melanogaster* spermatozoa [162]. Its immunological role in humans is seen by its deficiency in human leukocytes in which links to an increase occurs in the circulation of polymorphonuclear neutrophils that have diminished bactericidal activity [163]. Serum free hemocytes of the Eastern oyster, *Crassostrea virginica*, selectively enhance the release of α-mannosidase activity in presence of *E. coli* but not in presence of *Micrococcus roseus* and *Klebsiella oxytoca* [164]. The amino acid sequence of α-mannosidase in *Spodoptera frugiperda* Sf9 exhibits 35–57% homology with mammalian α-mannosidase II [165]; however, there are differences in catalytic activities between the two enzymes [166], which imply a diverse spectrum of biochemical activities that may account for the various protective roles of the enzyme.

α-glucosidase was released from the Md66, Md108, and UA-Md221 cell lines in the culture medium. Two hemocyte cell lines, NIAS-MaBr-92 and NIAS-MaBr-93, from *M. brassicae* demonstrate α-glucosidase activity in MTCM-1601 culture medium in which sucrose was included [167], implying that these lepidopteran hemocytes are capable of sucrose digestion [167,168]. The enzyme was not detectable in UA-Md203, which may indicate this cell line uses other molecules as carbon and energy sources. A similar absence of the enzyme occurred for *B. glabrata* hemocytes when activity of the enzyme was measured with photometric assays; however, α-glucosidase was readily apparent using polyacrylamide gels [160].

Strongly adherent cell lines UA-Md221 and UA-Md203 release more β-glucosidase than mildly and non-adherent cell lines to their culture medium. β-glucosidase released by hemocytes contributes to snail immunity against *S. mansoni* by increasing its activity in the plasma altering the parasite integument [159].

Investigating the interactions of insect hemocytes with microorganisms in a tissue culture system provides a unique type of information: hemocytes, while reacting to polystyrene, proliferate at the same time, a situation that is difficult to study in vivo. Floating Md66 and adhesive Md108 hemocyte lines were selected for bacterial response testing due to their shared primary culture origin and similar temperature optima, allowing each to serve as a control for the other.

Collectively, the APIZYM enzyme discharge data imply that *M. disstria* cell lines respond to polystyrene and *B. subtilis* by releasing possible antimicrobial enzymes. In PBS with *B. subtilis* as opposed to bacterial free PBS the activities of numerous enzymes of both cell lines rapidly increased while interacting with the selected antigens, supporting the contention that the enzymes may be part of effector mechanisms of non-self-responses by the adhering Md108 and the floating Md66 hemocyte cell line as either direct anti-antigen killing agents or indirect suppliers of nutrients/energy for hemocyte antigen reactions, e.g., phagocytosis.

Acid phosphatase, which was highly active in PBS supernatants of Md66 cells without bacteria, exhibited higher activity in presence of *B. subtilis*. The enzyme is an extensively studied lysosomal enzyme found in immunoreactive granular cells of insects [169] and granular and hyaline hemocytes of the decapods crustaceans *Homarus americanus*, *Panulirus interruptus*, and *Loxorhynchus grandis* [170] and in the hemocytes of the mollusk *Crassotrea madrasensis* [171]. Ultrastructure and cytochemical studies on *Galleria mellonella* hemocytes demonstrate that lysosomal particles with acid phosphatase are released when blood cells attempt to engulf latex beads [140]. The acid phosphatase levels in hemolymph plasma of the flesh fly, *Neobellieria bullata*, larvae represent secretion from two tissues: the fat body and hemolymph [172], implying that during an in vivo immune challenge of the insect larvae with antigens, acid phosphatase activity may have several origins. Similar conclusions were obtained for the origin of lysosomic enzymes in the hemolymph from the fat body in mollusks [136,139].

## 5. Conclusions

Morphological analysis, cell type frequencies, and enzyme activity analyses across four hemocytic cell lines (Md108, Md66, UA-Md221 and UA-Md203) reveal distinct metabolic signatures that differentiate these samples into meaningful groups. The UA-Md lines exhibit particularly distinctive enzyme profiles compared with Md108 and Md66, indicating different metabolic phenotypes or adaptation strategies. UA-Md cell lines emphasize protein degradation and complex carbohydrate processing, whereas Md lines follow distinct metabolic pathways, with Md66 exhibiting elevated esterase and phosphatase activities relative to Md108.

Dead *B. subtilis* elicited innate and metabolic responses in Md66 cells through a complex hierarchical enzyme cascade. Initial membrane-level signaling involved immediate phosphatase and esterase activation, followed by protein-processing enzymes and culminating in carbohydrate-degrading enzymes for metabolic-immunological adaptation. These temporal patterns suggest potential biomarkers: phosphatases and esterases for early bacterial detection, aminopeptidases for monitoring host–microbe interaction progression, and glycosidase patterns for metabolic assessment.

This work revealed that the addition of fetal bovine serum, as an ingredient in tissue culture medium, creates potential links with an advanced biological role on ex situ hemocytic physiology. Thermal responses and optimum temperature ranges of cell lines are dependent on the maternal tissue geographic origin collection site, which reflects the spatio-genetic origin attributes and mitochondrial cytochrome oxidase signatures of the local populations from which insects were collected. Serum-free hemocytes in PBS demonstrated non-self-recognition responses to polystyrene and bacteria through quantitative and qualitative alterations in their secretory enzyme profiles. The performance of semi-quantitative assessments of released enzymes by hemocytic cell lines can provide valuable insights into the time window of each enzyme release, revealing immune and metabolic signaling patterns.

## Figures and Tables

**Table 1:** Descriptive information for the Md66 hemocytic cellular forms detected in the culture medium at their optimum temperature.

Hemocyte Type	Description	Cell Size (μm) ^a^
**1**	Round cells, smooth edged with large central nucleus (~8 μm diameter)	7–24
**2**	Oval cells with large central nucleus (~8 μm diameter), chromatin not visible, filopodia with some membrane extension beyond the main cell mass and between the filopodia (arrow)	8–13
**3**	Elongated cells with one or more thick projections at one or both ends without or with an apical membrane marginalized with numerous filopodial structures; central nucleus with or without discernible chromatin	9–17
**4**	Oval cell with nucleus lacking discernible chromatin	13–20
**5**	Oval-trapezoid plasmatocyte-like cell with or without thick projections and with an occasional crenated membrane opposite the projections (arrow)	8–10
**6**	Elongated cell containing 5–6 spherical inclusions (~5–4 μm diameter)	9–13

^a^ Maximum dimensions (µm).

**Table 2:** Descriptive information for the Md108 hemocytic cellular forms detected in the culture medium at their optimum temperature.

Hemocyte Type	Description	Cell Size (μm) ^a^
**1**	Spherical cell with a large central nucleus (~8 μm diameter) and phase bright granules in the cytoplasm	4–12
**2**	Oval-oblong hemocytes with more than one projection (arrow), no discernible nucleus; phase dark with few granules	7–13
**3**	Oval cell with filopodia extending beyond the plasmacytoid membrane, ≥2 nuclei (5–8 μm diameter)	9–12
**4**	Oval granulated hemocyte, no detectable nucleus	5–7
**5**	Spherical cels with small eccentric nucleus (1 μm diameter; arrow)	4

^a^ Maximum dimensions (µm).

**Table 3:** Descriptive information for the UA-Md221 hemocytic cellular forms detected in the culture medium at their optimum temperature.

Hemocyte Type	Description	Cell Size (μm) ^a^	
**1**	Spherical cell with a large central nucleus.	7–12	
**2**	Pear-shaped cells with more than one lobopodial projection (arrow), nucleus, cytoplasm phase dark with few granules	10–14	
**3**	Stellate plasmatocyte with clearly visible nucleus in the center	17–22	
**4**	Oval, granulated cell, no detectable nucleus. A similar but smaller cell type was observed in Md108 (Md108 cell type 4).	13–20	
**5**	Granular cell with filopodial projections (arrow)	7–16	
**6**	Round cell with monopolar projection, which ends with dendritic-type tips	17–22	
**7**	Elongated, polarized cell with one (or more) stylet-type projections (arrow) on one side and numerous projections on the other. Large nucleus (3–4 μm) is visible in the center.	(24–31) × (5–8)	
**8**	Polymorphic elongated cell with one side oval shaped and the other ending in globular projections (arrow)	14–18	
**9**	Multifilopodial cell with round central body and peripheral globular condensations	16–24	
**10**	Oval cell with flat projected cytoplasmic extensions at both tips	19–26	
**11**	Multi-branched cell with asymmetrical radiations	22–34	
**12**	Polygonal cell shaped main cell with enlarged lobopodia	15–17 body 7–8 projection	
**13**	Dendritic hemocyte, with a linear axis	8–12 main; 15–17 body axis	
**14**	“Triploid” stellate cell, bifurcated, with linear filopodia	24–29	

^a^ Maximum dimensions.

## Data Availability

The original contributions presented in this study are included in the article. Further inquiries can be directed to the corresponding author.
